# The Biotherapeutic Potential of *Lactobacillus reuteri* Characterized Using a Target-Specific Selection Process

**DOI:** 10.3389/fmicb.2020.00532

**Published:** 2020-04-15

**Authors:** Valeria Sagheddu, Francesca Uggeri, Luisella Belogi, Laura Remollino, Paola Brun, Giulia Bernabè, Giancarlo Moretti, Andrea Porzionato, Lorenzo Morelli, Ignazio Castagliuolo, Marina Elli

**Affiliations:** ^1^AAT-Advanced Analytical Technologies S.r.l., Fiorenzuola d’Arda, Italy; ^2^Moviscom S.r.l., Rome, Italy; ^3^Nòos S.r.l., Rome, Italy; ^4^Department of Molecular Medicine, University of Padua, Padua, Italy; ^5^Department of Neurosciences, University of Padua, Padua, Italy; ^6^Department for Sustainable Food Process – DiSTAS, Università Cattolica del Sacro Cuore, Piacenza, Italy

**Keywords:** probiotic, lactobacilli, pathogen, intestine, gut, diarrhea

## Abstract

A growing body of clinical and experimental data supports the view that the efficacy of probiotics is strain-specific and restricted to particular pathological conditions, which means that newly isolated probiotic strains need to be targeted to a specific disease. Following national and international guidelines, we used a conventional *in vitro* experimental approach to characterize a novel strain of *Lactobacillus reuteri*, LMG P-27481, for safety (sensitivity to antibiotics and genome analysis) and putative efficacy (resistance to gastro-intestinal transit, adhesiveness, induction of cytokines, and release of antimicrobial metabolites). *In vitro* assays, which were carried out to examine the probiotic’s effect on diarrhea (lactose utilization, inhibition of pathogens such as bacteria and Rotavirus), showed that it was more efficacious with respect to well-known reference strains in antagonizing *Clostridioides difficile* (CD). Data confirming that the probiotic can effectively treat CD colitis was gained from *in vivo* trials involving mice conditioned with large spectrum antibiotics before they were subjected to CD challenge. Two out of the three antibiotic-treated groups received daily LMG P-27481 for different time durations in order to simulate a preventive approach (LMG P-27481 administered prior to CD challenge) or an antagonistic one (LMG P-27481 administered after CD challenge). Both approaches significantly reduced, with respect to the untreated controls, CD DNA concentrations in caecum and *C. difficile* toxin titers in the gut lumen. In addition, LMG P-27481 supplementation significantly mitigated body weight loss and the extent of inflammatory infiltrate and tissue damage. The study results, which need to be confirmed by *in vivo* clinical trials, have demonstrated that the *L. reuteri* LMG P-27481 strain is a promising probiotic candidate for the treatment of CD infection.

## Introduction

A variety of studies have evaluated the efficacy of probiotics in treating diarrhea, which represents a potentially serious health problem with more than 4 billion cases reported world-wide every year. McFarland et al. (2018) carried out an extensive systematic meta-analysis of the results of randomized controlled trials examining the use of probiotics to treat different types of diseases. In accordance with Ouwehand (2017), the investigators confirmed the strain- and disease-specificity of probiotics in treating diarrhea. Despite a wide heterogeneity across clinical studies, some probiotics have been found to be effective in treating antibiotic-associated adult diarrhea, *Clostridium difficile* diarrhea (CDAD), acute Rotavirus diarrhea, radiation therapy-related diarrhea, and enteral nutrition-related diarrhea. Their efficacy in treating traveler’s diarrhea (TD) has not, however, been supported by clinical data (Williams and Adcock, 2018). Hypothetical mechanisms explaining probiotics’ effects include competitive inhibition of pathogens, stabilization of the resident microbiota, and attenuation of increased gut barrier permeability. Their beneficial effects in terms of reducing the duration and intensity of fever has been demonstrated in children although they did not seem to affect the number of daily evacuations and accompanying symptoms such as vomiting (Salari et al., 2012).

The efficacy of probiotics in adults for the different types of diarrhea listed above has not yet been convincingly established and still requires further investigation (Ritchie and Romanuk, 2012; Salari et al., 2012). When Szajewska et al. (2019) examined in a systematic review 15 randomized controlled trials investigating the efficacy of *Lactobacillus rhamnosus* GG (LGG) in children affected by acute gastroenteritis, they concluded that the probiotic reduced the duration of diarrhea and hospitalization. Conflicting findings, which may be explained by the heterogeneity of the patients studied and methodological limitations, have nevertheless been reported. Capurso (2019) likewise reviewed data examining the use of *L. rhamnosus* GG supplementation to treat gastrointestinal infections and diarrhea, antibiotic and *C. difficile* associated diarrhea as well as its use for other pathological conditions.

Belonging to the *Lactobacillus* genus, *Lactobacillus reuteri* represents a natural inhabitant of the human and animal gastro-intestinal tract. It is an obligatory heterofermentative Lactobacillus that in particular conditions leads to the formation of carbon dioxide, ethanol, acetic acid and lactic acid following sugar fermentation. *L. reuteri* can also produce 3-hydroxypropionaldehyde (reuterin), a bacteriocin with antimicrobial properties, as well as folate and cobalamin (Mu et al., 2018). Some *L. reuteri* strains, such as DSM 17938 and RC-14, are already present in many commercially available food supplements. Evidence confirming the efficacy of *L. reuteri* in treating gastrointestinal disorders such as infantile colic, regurgitation, functional constipation, abdominal pain, and necrotizing enterocolitis (Srinivasan et al., 2018) has been extensively outlined in the literature.

Using the FAO/WHO (2002) for the evaluation of probiotics in food, whose timeliness has been confirmed by an expert panel (Hill et al., 2014; Shewale et al., 2014), the current study set out to evaluate the efficacy of a new probiotic strain in treating *C. difficile-*associated diarrhea. The new strain, named LMG P-27481, meets the conventional safety criteria for probiotics which is based on their survival in the GI tract as well as their ability to adhere to gut mucosa, to counteract the activity of pathogens and to boost the immune system. Its safety profile for human use was also assessed following the most recent international guidelines (EFSA Scientific Opinion, 2018). Characterization to assess the strain’s efficacy was, moreover, performed using an *in vivo* murine model of CD-associated diarrhea to investigate its ability to counteract the pathogen.

## Materials and Methods

### Identification of *L. reuteri* LMG P-27481

The LMG P-27481 *L. reuteri* strain was isolated in 2013 from a human fecal sample of a 9-month old vaginally delivered healthy infant. Written informed consent was obtained from the parents. The strain was identified at the genus level and then as a distinct *L. reuteri* species. LMG P-27481 was compared to other *L. reuteri* strains available on the Italian market to assess its originality using strain-specific REP-PCR amplification (Versalovic et al., 1994). Other relevant well-known commercial strains such as *L. reuteri* RC-14, L. *reuteri* DSM 17938, and *L. rhamnosus* ATCC 53103 were used as references for the assays conducted during the study (Mogna et al., 2012).

### Safety Assessment

Minimum inhibitory concentrations (MICs) of 15 different antibiotics were determined for the strain under investigation in accordance with ISO 10932:2010 (IDF 223:2010). MIC values were compared with European Food Safety Authority (EFSA) breakpoints reported in their Scientific Opinions 2012 and 2018. The tests were carried out using antibiotics and doses listed in Table 1. Contemporaneously, we excluded the presence of plasmids within the cell using the method of plasmid extraction outlined by Anderson and McKay (1983) and by sequencing the entire genome of LMG P-27481 strain in order to check its safety profile.

**TABLE 1:** The range of antibiotic concentrations used to determine the minimum inhibitory concentrations.

Antibiotic	Concentration range (μg/ml)
Gentamicin	0.5–256
Kanamycin	2–1024
Streptomycin	0.5–256
Neomycin	0.5–256
Tetracycline	0.12–64
Erythromycin	0.016–8
Clindamycin	0.03–16
Chloramphenicol	0.12–64
Ampicillin	0.03–16
Vancomycin	0.25–128
Virginiamycin (Quinupristin-Dalfopristin)	0.016–8
Linezolid	0.03–16
Trimethoprim	0.12–64
Ciprofloxacin	0.25–128
Rifampin	0.12–64

### Complete Genome Sequencing of *L. reuteri* LMG P-27481 Strain

Genomic DNA from an overnight culture of LMG P-27481 *L. reuteri*, which was extracted using the MasterPure Gram Positive DNA Purification kit (Epicenter, Cambio, United Kingdom) following the manufacturer’s instructions, was quantified utilizing NanoDrop^TM^ spectrophotometer A280/260 (Thermo Fisher Scientific, Milan, Italy). Total DNA sequencing was determined by GenProbio Srl (Parma, Italy) using a MiSeq Illumina platform. Genomic libraries were constructed employing the TruSeq DNA PCR-Free LT Kit (Illumina) using 2.5 μg of genomic DNA, which was fragmented using a Bioruptor NGS ultrasonicator (Diagenode, United States), and size was assessed using Agilent 2200Tape Station (Agilent Technologies). Library samples were loaded onto a Flow Cell V3 600 cycles (Illumina) following the technical support guide instructions. Five hundred sequencing cycles resulted in an average reading length of approximately 250 nucleotides for both paired-end sequences.

### Bioinformatic Analysis of *L. reuteri* LMG P-27481 Genome

The version 4.0.2 MIRA program (Chevreux et al., 1999, Computer Science and Biology: Proceedings of the German Conference on Bioinformatics) was used for de-novo assembly of the LMG P-27481 *L. reuteri* genome sequence. Assembly resulted in 134 contigs with a total genome size of 2,016,419 bp using a MIRA v4.0.2 assembler (Chevreux et al., 1999), with a coverage of 70.1X, GC content of 38.8%, N_50_ 29849. Open Reading Frames (ORFs) prediction and subsequent GeneBank file construction were performed using Prodigal v2.6 software (Hyatt et al., 2010). Automatic annotation of the ORFs was carried out using a custom script performing a BLASTp search against the NCBI database (Milani et al., 2014). Ribosomal RNA gene prediction was performed using RNAmmer v1.2 (Lagesen et al., 2007), and transfer RNA gene prediction was carried out using tRNAscan-SE v1.21 (Lowe and Eddy, 1997).

Reordering of the final contigs comparable to NCBI template genomes *L. reuteri* DSM 20016 was performed using Mauve v2.3.1 (Darling et al., 2004). Burrows-Wheeler Alignment with SAMtools software (Li and Durbin, 2009) and VarScan v2.2.3 (Koboldt et al., 2013) were carried out to optimize the assembly to obtain final contigs. To confirm the results obtained from the MIC evaluation of the strain, the antibiotic resistome of LMG P-27481 *L. reuteri* was identified using Rapsearch (Zhao et al., 2012) against a custom database and CARD (McArthur et al., 2013) and Transporter Classification Database (TCDB) (Saier et al., 2014). Prediction of the mobilome was carried out using the PHAge Search Tool PHAST (Zhou et al., 2011) and the ISfinder database (Siguier, 2006). Prediction of the putative virulence genes was carried out using the Virulence Factors Database (VFDB) (Chen et al., 2005) and that of the bacteriocin encoding genes was performed using the Bagel3 software package (van Heel et al., 2013). Other relevant factors with a putative adhesion to probiotic functions, such as mucus and fibronectin-binding proteins and S-layer proteins, pilus encoding genes and those involved in Extracellular polysaccharide synthesis (EPS), were predicted using a custom database based on NCBI RefSeq (Pruitt et al., 2007).

### Conventional Characterization of *L. reuteri* LMG P-27481

The procedures described by Charteris et al. (1998) and Gilliland et al. (1984) were followed to investigate the ability of the newly isolated and reference *L. reuteri* DSM 17938 and *L. reuteri* RC-14 strains to survive the hostile conditions of the gastrointestinal environment including the high pH levels of gastric and duodenal juices. Their ability to adhere to the human mucosa was verified and quantified using cultured monolayers of HT-29 cells (Jensen et al., 2012). All the strains were cultured in MRS medium at 37°C in anaerobiosis and collected by centrifugation. Bacteria were resuspended in sterile DMEM at 10^8^ CFU/ml and added to HT-29 monolayers (MOI 1:50/cells to probiotic). The medium was incubated for 60 min at 37°C. Then, it was removed and discarded and sterile DMEM was added to the monolayers. The medium was checked three times to verify that all unbound bacteria were removed. One ml of sterile MRS was then added to each monolayer and the cells were collected using a cell scraper. The material was collected using a sterile syringe and passed three times through an 18-needle gauge, and following decimal dilutions, seeded on MRS agar to quantify the live bacteria. The adhesion was expressed as the percentage of viable bacterial cells adhering to HT-29. The experiments were performed in triplicate. *L. paracasei* ATCC 344 was used as the technical control.

### The *in vitro* Effect on Human Dendritic Cells

Following international guidelines for the characterization of the properties of probiotics, the immune modulatory potential of the LMG P-27481 strain was verified on human-derived dendritic cells (DCs). The DCs were generated from monocytes obtained from the peripheral blood of healthy donors and cultured in complete RPMI medium containing granulocyte-macrophage colony-stimulating factor (GM-CSF, 50 ng/mL; Peprotech) and interleukin-4 (20 ng/mL; Peprotech, Milan, Italy) for 6 days (Mileti et al., 2009). On the day of the experiment, the DCs were collected, washed by centrifugation, counted, and either incubated in medium alone or in medium without antibiotics containing the specified probiotic strain (MOI 1:10/DC to probiotic) obtained from a fresh culture in the logarithmic growth phase. The dendritic cells were incubated 1 h at 37°C, then collected, pelleted by centrifugation, resuspended in 1 ml of sterile RPMI and subjected to centrifugation (800 rpm × 10 min). The cells were then incubated in fresh complete medium containing gentamicin (100 μg/ml). After 24 h, the DCs were collected by centrifugation, IL-10 and IL12p70 were quantified in the culture supernatants by ELISA (eBioscience), and the anti-inflammatory index was calculated (IL-10/IL12p70 concentration ratio).

### The Release of Hydrogen Peroxide

The ability to release H_2_O_2_ in the surrounding environment confers antimicrobial and preservative properties to some lactobacilli. Bacterial strains were grown in MRS broth to assess the LMG P-27481 strain’s ability to release H_2_O_2_. The cultures were then centrifuged, washed twice, and finally resuspended with sodium phosphate buffer containing glucose 5 mM. After being incubated in refrigerated conditions for a maximum of 24 h, the cultures were centrifuged, and the supernatants were assayed for H_2_O_2_. Hydrogen peroxide was quantified following the protocol of Yap and Gilliland (2000). The RC-14 and DSM 17938 strains were used as positive and negative controls, respectively.

### Reuterin Secretion

Some strains of *L. reuteri* are known to produce broad-spectrum antimicrobial substance, reuterin (Talarico et al., 1988), a product of the glycerol metabolism that is active against several intestinal pathogens. The ability to produce this bacteriocin was verified using the colorimetric test described by Cadieux et al. (2008) on a 24-h culture of the strain grown in MRS medium supplemented with glycerol (250 mM final concentration). The principle behind the test is that of transforming glycerol into 3-hydroxypropionaldehyde (reuterin) and to quantify it using a spectrophotometric method at 560 nm (Doleyres et al., 2005; Ortiz-Rivera et al., 2017).

### Lactose Metabolism

The ability to use lactose as the growth substrate was tested by inoculating washed pure suspensions of *L. reuteri* DSM 17938, *L. rhamnosus* ATCC 53103, and *L. reuteri* LMG P-2748 in MRS medium without dextrose, containing either 1% glucose or 1% lactose as the sole carbon source (Arnold et al., 2018). After being incubated for 18 h in microaerophilic conditions at 37°C, live bacteria were enumerated by seeding decimal dilution of the cultures on MRS agarose plates. T18 results were compared to the initial counts at T0 for the different carbohydrate sources.

### The *in vitro* Effect on Intestinal Pathogen Growth

These experiments were designed to assess the ability of the LMG P-27481 strain to exert a direct inhibitory effect on some of the most common intestinal pathogens. The pathogen strains used for these tests were obtained from ATCC and from the Culture Collection of the University of Göteborg (Sweden). The two *L. reuteri* strains (LMG P-27481 and DSM 17938) and *L. rhamnosus* ATCC 53103 were cultured in MRS medium, *E. coli* and *Salmonella enterica* in Luria Bertani (LB) broth, and *C. difficile* in Brain Heart Infusion (BHI) broth. To test the antimicrobial activity, the lactobacilli were separately inoculated at 10^7^ CFU/ml and grown in MRS broth anaerobically at 37°C for 24 h. Once the bacteria had grown, the cultures were centrifuged, the supernatants were sterile filtered (0.2 μm), and the pH corrected to 7.0 before being inoculated with *E. coli*, *Salmonella* or *C. difficile* (10^7^ CFU/ml) and incubated at 37°C. The aliquots of bacterial cultures were collected and plated 6, 12, and 24 h after inoculation onto appropriate agar media to quantify the viable pathogens. Each experiment was performed three times with triplicate determinations.

### Th*e in vitro* Effect on the Rotavirus Infection

Rotavirus (ATCC VR-2018) was expanded and quantified on MA-104 (rhesus monkey kidney) cells. The efficacy of the *L. reuteri* LMG P-27481 strain in treating the Rotavirus infection was assessed utilizing two *in vitro* HT-29 cells protocols mimicking the human intestinal epithelium. In the pre-treatment *in vitro* model the strains were placed in contact with HT-29 cells (MOI 1:50/cells to probiotic) prior to exposure to trypsin activated human rotavirus (Guerrero et al., 2000). Seventy-two hours later, the cells were collected, and total RNA was extracted. In the competition protocol, trypsin activated human rotavirus was pre-incubated with the different probiotic strains for 90 min. The bacteria + virus mixture was briefly centrifuged, and then the clear supernatant was used to infect confluent monolayers of human intestinal epithelial HT-29 cells. Seventy-two hours later, the cells were collected, total RNA was extracted using the SV Total RNA Isolation System (Promega), and reverse-transcribed into complementary DNA. Viral genome copies were quantified with SYBR Green PCR Master Mix in an ABI PRISM 7000 Sequence Detection System (Applied Biosystems) using specific primers (van Maarseveen et al., 2010).

### The *in vivo* Inhibitory Effect on *C. difficile* Infection

**FIGURE 1:**
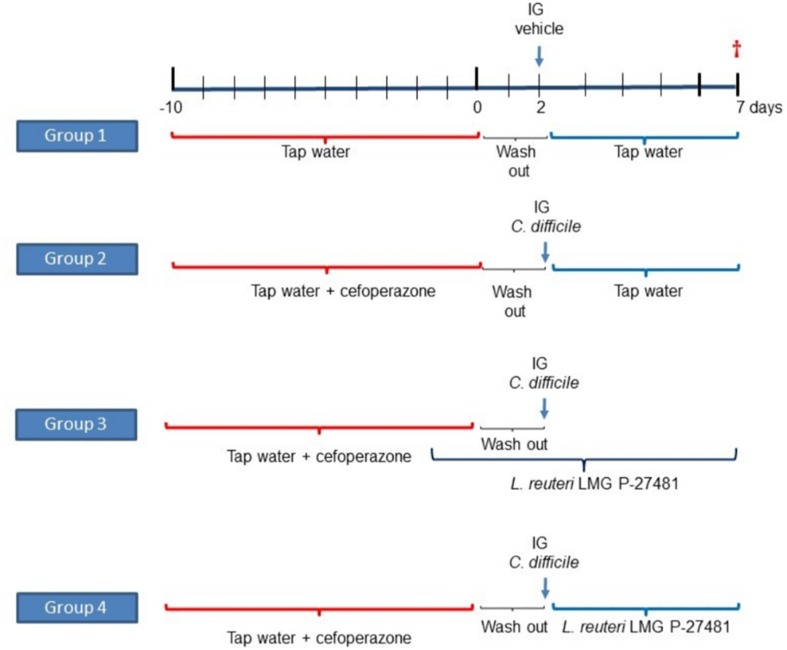
The experimental protocol of *L. reuteri* LMG P-27481 treatment of *C. difficile* colitis in mice.

LMG P-27481‘s ability to inhibit *C. difficile* infection was assessed *in vivo* by verifying intestinal colonization and inflammation in mice treated with cefoperazone (Theriot et al., 2011). Twenty-four adult C57Bl/6 mice, purchased from Envigo Srl (Udine, Italy), were randomly allocated to one of four experimental groups. Experimental group 1 was not subjected to antibiotic treatment and did not receive LMG P-27481; experimental groups 2, 3, and 4 received a broad-spectrum antibiotic (cefoperazone 0.5 mg/ml) in drinking water for 10 days. Two days after the antibiotic was discontinued mice received an intragastric suspension of *C. difficile* VPI strain 10463 (10^5^ CFUs) grown in BHI broth in anaerobic conditions. Experimental group 2 received only the *C. difficile* challenge without LMG P-27481 treatment. Experimental group 3 was administered an intragastric suspension of *L. reuteri* LMG P-27481 (10^9^ CFU/day) 2 days before the antibiotic administration, which lasted for 9 days. Experimental group 4 received *L. reuteri* LMG P-27481 (10^9^ CFU/day) beginning on day 1 after *C. difficile* challenge, that continued for 4 days (Figure 1).

Body weight was monitored daily until the animals were sacrificed 5 days after the *C. difficile* challenge. The caecum content was collected to assess the presence of *C. difficile* toxins, diluted 1:1 (vol/weight) with sterile PBS, and centrifuged at 10,000 × *g* for 5 min. The clear supernatant was then sterile filtered (0.2 μm) and serially diluted on Vero cell monolayers to determine the cytotoxin titer, which was defined as the highest dilution needed to cause >50% rounding of all cells. *C. difficile* colonization was evaluated by seeding aliquots of caecal content on *C. difficile* selective Agar (CDSA) plates (Becton Dickinson) incubated at 37°C in anaerobiosis. In addition, total DNA was extracted from 200 mg of caecal content using QIAmp DNA Stool mini extraction kit (Qiagen), and purified DNA was used as a template for qPCR using *C. difficile* 16S specific primers (Truong et al., 2017). Data were quantified using the ΔΔCt method and 16S rDNA as the reference gene. Tissue specimens from the colon of the mice were collected immediately after they were sacrificed and either fixed in 4% buffered formalin or snap frozen in liquid nitrogen. After the tissues had been kept in formalin for 24 h, they were paraffin embedded. At that time, 6 μm thick sections were cut, subjected to standard hematoxylin & eosin staining and scored for inflammatory damage by a pathologist (A.P.) (Castagliuolo et al., 1994). The frozen specimens were used to quantify myeloperoxidase activity (MPO) as a marker of neutrophil infiltration, as described elsewhere (Castagliuolo et al., 2005). All the experimental protocols were approved by the Animal Care and Use Ethics Committee of the University of Padua under license from the Italian Ministry of Health, and they were compliant with national and European guidelines for handling experimental animals and using them in experiments.

### Statistical Analysis

Data are reported as mean ± standard error of the mean (SEM) or mean ± standard deviation (SD). Statistical analysis was performed using GraphPad Prism Software 6.0 (GraphPad Software Inc., La Jolla, CA, United States). Student’s *t*-test was used to compare two independent groups; One-way ANOVA was used to compare more than two experimental group. *p* < 0.05 values were considered statistically significant.

## Results

### Identification and Probiotic Features of *L. reuteri* LMG P-27481

**FIGURE 2:**
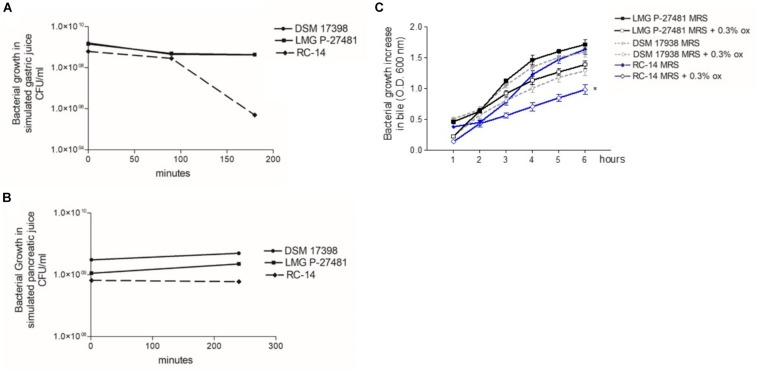
*Lactobacillus reuteri* LMG P-27481’s tolerance to *in vitro* gut transit gastric acid **(A)**, pancreatic juice **(B)** and bile **(C)** exposure. **(A)** The probiotic’s growth, expressed in CFU/ml, following 0, 90, and 180 min of exposure to simulated gastric juice (*n* = 3 replicates for each strain). **(B)** The probiotic’s growth, expressed in CFU/ml, following 0 and 240 min of exposure to simulated pancreatic juice (*n* = 3 replicates for each strain). **(C)** Growth increase of probiotics, expressed as optical density OD 600 nm, following exposure for 6 h to bile salts. There are two curves for each strain tested: the reference growth standard on conventional laboratory medium MRS and the test curve reflecting the same medium supplemented with 0.3% vol/vol Oxgall.

**FIGURE 3:**
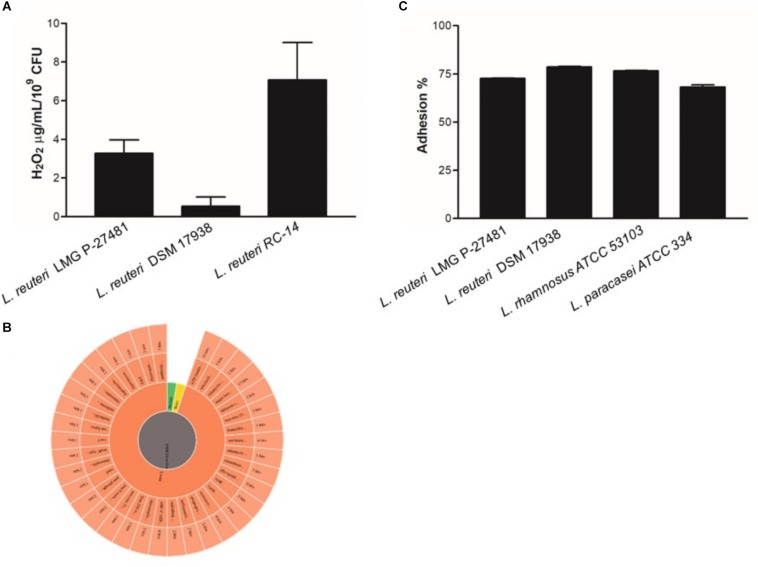
*Lactobacillus reuteri* LMG P-27481 hydrogen peroxide release and adherence to human HT-29 monolayers. **(A)** Production of H_2_O_2_ by probiotics expressed as μg/ml of metabolite released by 10^9^ viable CFUs after 24 h of incubation. **(B)** Antibiotic resistance image obtained by CARD showed the presence of nonperfect and strict and 111 loose hits. **(C)** Confluent HT-29 monolayers were washed and incubated with the probiotic strain (MOI 1:10) for 1 h at 37°C. To quantify adhering bacterial cells, the monolayers were extensively washed, lysed and aliquots seeded on MRS agar plates. Following 48 h of incubation in anaerobiosis, the colonies were enumerated and adhering bacteria expressed as the percentage of the total initial population of viable probiotic cells (*n* = 3 different experiments in duplicate). Data are presented as mean ± SD.

The LMG P-27481 strain was selected from other human isolates belonging to the *Lactobacillus reuteri* species in view of its susceptibility to antibiotics according to the breakpoint values for *L. reuteri* suggested by EFSA’s Scientific Opinion for 2018 (EFSA Scientific Opinion, 2018). *L. reuteri* species can be found, in fact, in the list of qualified presumption of safety (QPS) species, and our investigations using phenotypic and genotypic methods have confirmed the safety of LMG P-27481 strain. The strain was compared to other well-known, commercially available probiotic strains, such as *L. reuteri* DSM 17938, RC-14 and *L. rhamnosus* ATCC 53103 during our assessment and convincingly resulted a new potential probiotic product. We found that strain LMG P-27481 was able to survive exposure to the pH of the simulated gastric juice; it was comparable to DSM 17938 and higher than RC-14 strain (Figure 2A). Moreover, all the tested strains showed a marked resistance to exposure to simulated pancreatic juice (Figure 2B). After 6 h of incubation at 37°C, the presence of bile in the culture medium caused a comparable reduction in optical density in strains DSM 17938 and LMG P-27481; RC-14 strain showed instead a greater susceptibility (Figure 2C). LMG P-27481’s ability to produce oxygen peroxide placed it an intermediate level of the reference strains. In fact, 2.58 μg/10^9^ CFU was detected after 1-h of incubation and 3.96 μg/10^9^ CFU after 24 h (Figure 3A). The data obtained during the course of two different tests to determine the presence of reuterin in the culture medium were in agreement with previous studies uncovering reuterin release by DSM 17938 (positive control) and RC-14 (negative control) strains and the absence of reuterin in LMG P-27481 culture medium (data not shown).

### Genome Sequencing: The Safety and Functional Profile of the LMG P-27481 Strain

As reported by Saulnier et al. (2011) genome analysis of *L. reuteri* LMG P-27481 strain revealed features that are compatible with those of other *L. reuteri* strains. The average genome size for the *L. reuteri* species is approximately 2.0 Mbp with a total average number of 1.600 genes, and G+C content of 39%. Genome sequencing has been deposited and is available (WHOJ00000000 release public date 2020-02-03). The “unique” genes of *L. reuteri* LMG P-27481 are listed in Table 2. With regard to resistome detection, 15 putative antibiotic resistance genes with enzymatic function and 13 putative antibiotic resistance genes with efflux/transport function were identified. Graphical representation of antibiotic resistances obtained by CARD indicated the presence of 111 loose with the absence of perfect or strict hits underlying the safety of the *L. reuteri* LMG P-27481 (Figure 3B). Most of the sequences identified belonged to ß-lactamase enzymes, but those gene(s) in *L. reuteri* were considered non-transferable by several authors because of their chromosomal location. No other antibiotic resistance mechanisms were identified. *L. reuteri* LMG P-27481 MIC values are listed in Table 3. During the investigation for possible virulence genes, 38 sequences were sorted out by bioinformatic tools as predictors of virulence factors. However, based on strict BLASTP standard (90% identity cutoff) none of the sequences raised from the VFDB analysis were considered as significant reporting identity at most of 70.4% (Li et al., 2019). The investigation confirmed that the LMG P-27481 strain was safe given the absence of significant sequence identity with any well-known virulence genes. No S-layer putative proteins were found in the *L. reuteri* LMG P-27481 genome. We performed comparative genomics between the LMG P-27481 strain and 18 other *L. reuteri* genomes available in public databases (Table 4). The analysis uncovered 188 unique sequences for strain LMG P-27481; 81 of these were classified as hypothetical proteins of unknown function. Most of the remaining putative genes, such as mucus-binding proteins, EPS biosynthesis and cell wall anchor domains, seemed to be involved in cell surface design and therefore in the interaction with the host.

**TABLE 2:** “Unique” genes of *L. reuteri* LMG P-27481.

LMG_P-27481 ORFs	LMG_P-27481 annotation
LMG_P-27481_0045	Hypothetical protein
LMG_P-27481_0048	Hypothetical protein
LMG_P-27481_0205	Hypothetical protein
LMG_P-27481_0214	Type III restriction protein, res subunit
LMG_P-27481_0286	YSIRK signal domain/LPXTG anchor domain surface protein
LMG_P-27481_0293	Cytochrome C554
LMG_P-27481_0387	Transposase
LMG_P-27481_0432	Acetylornithine deacetylase
LMG_P-27481_0511	Hypothetical protein
LMG_P-27481_0514	Hypothetical protein
LMG_P-27481_0520	Cro/C1-type HTH DNA-binding domain-containing protein
LMG_P-27481_0523,LMG_P-27481_0840	Hypothetical protein
LMG_P-27481_0583	Oxalyl-CoA decarboxylase
LMG_P-27481_0650	Hypothetical protein
LMG_P-27481_0666	Competence protein CoiA
LMG_P-27481_0754	Hypothetical protein
LMG_P-27481_0846	Integrase
LMG_P-27481_0849	Hypothetical protein
LMG_P-27481_0850	Hypothetical protein
LMG_P-27481_0851	Alpha-amylase
LMG_P-27481_0852	Hypothetical protein
LMG_P-27481_0853	Hypothetical protein
LMG_P-27481_0854	Hypothetical protein
LMG_P-27481_0856	Glycosyl hydrolase family 25
LMG_P-27481_0869	Peptidoglycan-binding protein LysM
LMG_P-27481_0908	Hypothetical protein
LMG_P-27481_0909	Hypothetical protein
LMG_P-27481_0910	PHAGE protein, ATPase
LMG_P-27481_0914	Cro/Cl family transcriptional regulator
LMG_P-27481_0915	Hypothetical protein
LMG_P-27481_0919	Hypothetical protein
LMG_P-27481_0920	Hypothetical protein
LMG_P-27481_0921	Hypothetical protein
LMG_P-27481_0922	Hypothetical protein
LMG_P-27481_0923	Hypothetical protein
LMG_P-27481_0924	Hypothetical protein
LMG_P-27481_0927	Hypothetical protein
LMG_P-27481_0928	Phage protein
LMG_P-27481_0936	Hypothetical protein
LMG_P-27481_0937	DNA replication protein
LMG_P-27481_0938	Hypothetical protein
LMG_P-27481_0942	Hypothetical protein
LMG_P-27481_0943	Hypothetical protein
LMG_P-27481_0944	Chorismate synthase
LMG_P-27481_0946,LMG_P-27481_1265	Hypothetical protein
LMG_P-27481_0947	Hypothetical protein
LMG_P-27481_0948	Hypothetical protein
LMG_P-27481_0950	Hypothetical protein
LMG_P-27481_0952	Hypothetical protein
LMG_P-27481_0969	NLP P60 protein
LMG_P-27481_0970	Hypothetical protein
LMG_P-27481_0971	Hypothetical protein
LMG_P-27481_0972	Tail protein
LMG_P-27481_0973	Hypothetical protein
LMG_P-27481_0974	Hypothetical protein
LMG_P-27481_0975	Hypothetical protein
LMG_P-27481_0976	Hypothetical protein
LMG_P-27481_1014	Hypothetical protein
LMG_P-27481_1098	Cell wall anchor
LMG_P-27481_1185	Hypothetical protein
LMG_P-27481_1187,LMG_P-27481_2156	N-acetylmuramoyl-L-alanine amidase
LMG_P-27481_1193	Hypothetical protein
LMG_P-27481_1194	Polysaccharide biosynthesis family protein
LMG_P-27481_1195	Exopolysaccharide biosynthesis protein
LMG_P-27481_1196	Transposase
LMG_P-27481_1197	O-antigen polysaccharide polymerase Wzy
LMG_P-27481_1239	PTS sugar transporter subunit IIA
LMG_P-27481_1249	Chromosome segregation ATPase, partial
LMG_P-27481_1250	NLP/P60 protein
LMG_P-27481_1251	Phage tail protein
LMG_P-27481_1252	Tape measure protein
LMG_P-27481_1253	Hypothetical protein
LMG_P-27481_1254	Small major structural protein
LMG_P-27481_1255	Tail component protein
LMG_P-27481_1256	Tail component protein
LMG_P-27481_1257	Phage head-tail adaptor
LMG_P-27481_1297	Hypothetical protein
LMG_P-27481_1304	Hypothetical protein
LMG_P-27481_1455	Hypothetical protein
LMG_P-27481_1458	Hypothetical protein
LMG_P-27481_1459	Hypothetical protein
LMG_P-27481_1460	Hypothetical protein
LMG_P-27481_1461	Hypothetical protein
LMG_P-27481_1462	Acyltransferase
LMG_P-27481_1472	CDP-diacylglycerol diphosphatase
LMG_P-27481_1479	N-acetylmuramoyl-L-alanine amidase
LMG_P-27481_1491	Amino acid permease
LMG_P-27481_1525	Hypothetical protein
LMG_P-27481_1526	Transposase
LMG_P-27481_1527	Uracil-DNA glycosylase
LMG_P-27481_1541	Hypothetical protein
LMG_P-27481_1543	Hypothetical protein
LMG_P-27481_1651	NAD-dependent malic enzyme
LMG_P-27481_1671	ABC transporter related
LMG_P-27481_1735	MFS transporter
LMG_P-27481_1744	Amino acid permease
LMG_P-27481_1745	Decarboxylase
LMG_P-27481_1755	Hypothetical protein
LMG_P-27481_1762	Hypothetical protein
LMG_P-27481_1763	Hypothetical protein
LMG_P-27481_1764	Hypothetical protein
LMG_P-27481_1765	Short-chain dehydrogenase
LMG_P-27481_1767	Acetyltransferase
LMG_P-27481_1772	Hypothetical protein
LMG_P-27481_1785	CsbD family protein
LMG_P-27481_1846	Hypothetical protein
LMG_P-27481_1847	Hypothetical protein
LMG_P-27481_1855,LMG_P-27481_2303,LMG_P-27481_1454,LMG_P-27481_2232,LMG_P-27481_2259	Transposase
LMG_P-27481_1871	Hemolysin
LMG_P-27481_2037	Transcriptional regulator
LMG_P-27481_2073,LMG_P-27481_1453	Integrase, catalytic region
LMG_P-27481_2075	hypothetical protein
LMG_P-27481_2077	integrase
LMG_P-27481_2078	Hypothetical protein
LMG_P-27481_2079	Hypothetical protein
LMG_P-27481_2080	Hypothetical protein
LMG_P-27481_2081	DNA polymerase III
LMG_P-27481_2084	Hypothetical protein
LMG_P-27481_2085	Hypothetical protein
LMG_P-27481_2086	Hypothetical protein
LMG_P-27481_2087	Hypothetical protein
LMG_P-27481_2088	Hypothetical protein
LMG_P-27481_2089	XRE family transcriptional regulator
LMG_P-27481_2095	Hypothetical protein
LMG_P-27481_2101	Integrase
LMG_P-27481_2102	Transposase
LMG_P-27481_2103	LysR family transcriptional regulator
LMG_P-27481_2104	5-methyltetrahydropteroyltriglutamate–homocyste ine methyltransferase
LMG_P-27481_2105	5,10-methylenetetrahydrofolate reductase
LMG_P-27481_2107	Cell wall anchor protein
LMG_P-27481_2108	Mucus-binding protein
LMG_P-27481_2137	Hypothetical protein
LMG_P-27481_2138	DNA-binding protein
LMG_P-27481_2139	Restriction endonuclease
LMG_P-27481_2140	DNA methylase N-4/N-6 family protein
LMG_P-27481_2141	Hypothetical protein
LMG_P-27481_2142	Hypothetical protein
LMG_P-27481_2143	Hypothetical protein
LMG_P-27481_2144	Hypothetical protein
LMG_P-27481_2145	Hypothetical protein
LMG_P-27481_2146	ATPase para family protein
LMG_P-27481_2147	Phage integrase
LMG_P-27481_2161	MFS transporter, partial
LMG_P-27481_2163	K02314 replicative DNA helicase
LMG_P-27481_2165	hypothetical protein
LMG_P-27481_2166	Guanine permease
LMG_P-27481_2167	Adenine phosphoribosyltransferase
LMG_P-27481_2168	Hypothetical protein
LMG_P-27481_2173	Glycerol-3-phosphate transporter
LMG_P-27481_2186	Hypothetical protein
LMG_P-27481_2187	Restriction endonuclease
LMG_P-27481_2189,LMG_P-27481_2317,LMG_P-27481_0403,LMG_P-27481_2213,LMG_P-27481_0570,LMG_P-27481_0092,LMG_P-27481_0775,LMG_P-27481_0839,LMG_P-27481_2016,LMG_P-27481_2134,LMG_P-27481_1225,LMG_P-27481_2282,LMG_P-27481_1076,LMG_P-27481_1045,LMG_P-27481_1996,LMG_P-27481_0115,LMG_P-27481_1661,LMG_P-27481_0752,LMG_P-27481_0105,LMG_P-27481_2185,LMG_P-27481_0344,LMG_P-27481_0170,LMG_P-27481_0119,LMG_P-27481_0522,LMG_P-	
27481_1775,LMG_P-27481_1107,LMG_P-27481_2274,LMG_P-27481_1908,LMG_P-27481_1770,LMG_P-27481_0532,LMG_P-27481_2288,LMG_P-27481_1190,LMG_P-27481_1863,LMG_P-27481_1243,LMG_P-27481_2300,LMG_P-27481_1487,LMG_P-27481_1710,LMG_P-27481_0409,LMG_P-27481_2319,LMG_P-27481_0645,LMG_P-27481_0196	Hypothetical protein
LMG_P-27481_2195	2-amino-4-hydroxy-6-hydroxymethyldihydropteridin e pyrophosphokinase
LMG_P-27481_2198	Bifunctional folylpolyglutamate synthase/dihydrofolate synthase
LMG_P-27481_2204	Phage integrase
LMG_P-27481_2206	Hypothetical protein
LMG_P-27481_2208	Glycerol-3-phosphate transporter
LMG_P-27481_2212	AI-2E family transporter
LMG_P-27481_2222	2,5-diketo-D-gluconic acid reductase
LMG_P-27481_2223	Restriction endonuclease
LMG_P-27481_2225,LMG_P-27481_2159	Restriction endonuclease
LMG_P-27481_2228	Multidrug ABC transporter permease
LMG_P-27481_2239,LMG_P-27481_2183	YSIRK signal domain/LPXTG anchor domain surface protein
LMG_P-27481_2240	Acetylornithine deacetylase
LMG_P-27481_2242	Glycerol-3-phosphate transporter
LMG_P-27481_2243,LMG_P-27481_2162	hypothetical protein
LMG_P-27481_2244,LMG_P-27481_2205	K02314 replicative DNA helicase
LMG_P-27481_2245	Phospholipase
LMG_P-27481_2248	MazF family toxin-antitoxin system
LMG_P-27481_2250	3-beta-hydroxysteroid dehydrogenase
LMG_P-27481_2253	Glycerol-3-phosphate transporter
LMG_P-27481_2257	Ribonuclease HII
LMG_P-27481_2272	Bacterial lipoprotein
LMG_P-27481_2276	Integrase
LMG_P-27481_2278	Carbamoyl-phosphate synthase large chain
LMG_P-27481_2286	Guanine permease
LMG_P-27481_2287	Hypothetical protein
LMG_P-27481_2292	DNA polymerase III subunit gamma/tau
LMG_P-27481_2293	Two-component system sensor histidine kinase
LMG_P-27481_2296	Farnesyl pyrophosphate synthetase
LMG_P-27481_2297	Non-canonical purine NTP pyrophosphatase
LMG_P-27481_2305	Glutamine amidotransferase
LMG_P-27481_2307	30S ribosomal protein S4
LMG_P-27481_2313	UDP-glucose 4-epimerase
LMG_P-27481_2315	Asparagine–tRNA ligase
LMG_P-27481_2325	Transposase
LMG_P-27481_2327	Peptide ABC transporter substrate-binding protein
LMG_P-27481_2338	Transposase
	

**TABLE 3:** *Lactobacillus reuteri* LMG P-27481 Minimum inhibitory concentration values.

Antibiotic	*L. reuteri* LMG P-27481 (μg/ml)	EFSA cut-off 2018 for *L. reuteri* (μg/ml)
Gentamycin	4	16
Kanamycin	64	64
Streptomycin	32	64
Tetracycline	16	32
Erythromycin	0,5	1
Clindamycin	0,063	4
Chloramphenicol	4	4
Ampicillin	1	2
Vancomycin	>128	n.r.
Neomycin	4	/
Virginiamycin	1	/
Ciprofloxacin	64	/
Linezolid	4	/
Rifampicin	0,125	/
Trimethoprim	64	/

*n.r. not requested, / not reported.*

**TABLE 4:** Comparative genomics analyses performed with 18 *L. reuteri* publicly available strains. The percentages of identity with *L. reuteri* LMG P-27481 are reported.

	*L. reuteri* ATCC53608	*L. reuteri* JCM1112	*L. reuteri* LTH5448	*L. reuteri* I5007	*L. reuteri* ZLR003	*L. reuteri* TMW1.656	*L. reuteri* TD1	*L. reuteri* MM2-3	*L. reuteri* DSM20016	*L. reuteri* mlc3	*L. reuteri* 100-23	*L. reuteri* TMW1.112	*L. reuteri* lpuph	*L. reuteri I*RT	*L. reuteri* LTH2584	*L. reuteri* SD2112	*L. reuteri* MM4-1A	*L. reuteri* CF48-3A
***L. reuteri* LMG P-27481**	96,67	98,88	96,65	96,76	96,69	96,2	96,53	98,86	98,86	95,68	96,35	96,14	96,53	98,92	96,18	96,33	98,87	96,12

### *L. reuteri* LMG P-27481 Efficiently Adheres to Human Intestinal Epithelial Cells

In terms of adhesive capacity to the HT-29 cell line, a high percentage of viable cells of the reference strains adhered to human enterocytes although with a slightly different aptitude (Figure 3C). As expected, the *L. rhamnosus* ATCC 53103 and DSM 17938 reference strains showed the highest robust adhesion ability (79 and 78% on average, respectively); *L. paracasei* ATCC 334 was the least effective (adhesion ranging between 67 and 71%). Indeed, *L. reuteri* strain LMG P-27481 adhered efficiently to human intestinal epithelial cells since it was only slightly less adhesive (73%) with respect to the most efficient strains.

### *L. reuteri* LMG P-27481 Induces a Distinctive Cytokine Profile in Human DC

**FIGURE 4:**
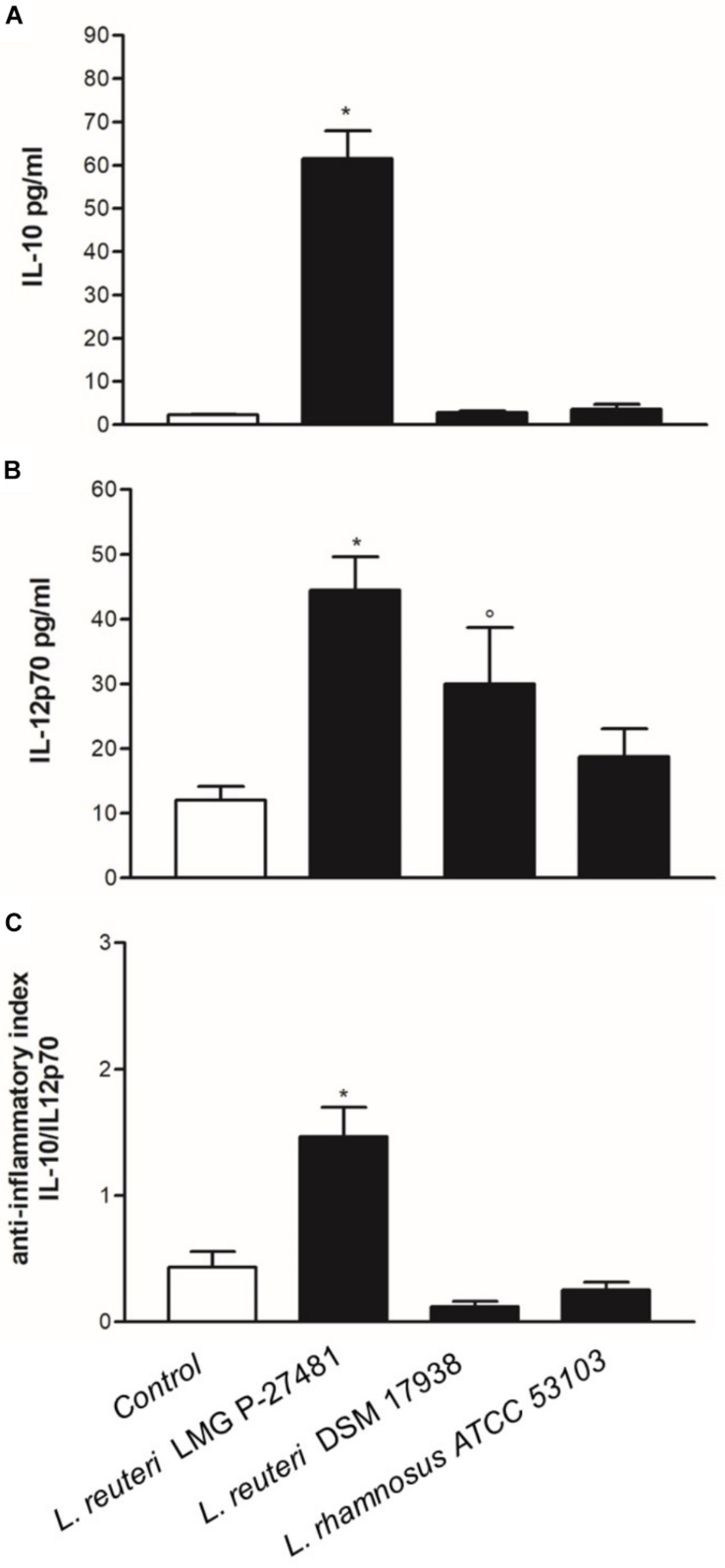
*Lactobacillus reuteri* LMG P-27481 induces an anti-inflammatory cytokine profile in human dendritic cells. The DCs obtained from differentiated peripheral blood monocytes were incubated (1:10 ratio) with the reported live bacterial strains for 1 h in medium without antibiotics. The cells were then collected, washed by centrifugation and incubated for an additional 23-h period in complete medium with antibiotics. Culture supernatants were collected. **(A)** IL-10 content quantified by ELISA. **(B)** IL-12p70 concentration assessed by ELISA. **(C)** Shows the anti-inflammatory index calculated as the ratio between IL-10 and IL-12p70 listed in Panels **(A,B)**. Data are reported as mean ± SD on values obtained on 3 different donors. *P < 0.05 versus control and °*P* < 0.05 versus LMG P-27481.

We exposed human immature DCs obtained from peripheral blood monocytes to viable logarithmic-phase lactobacilli and quantified the cytokines in the culture medium to determine the effect of our strain on DC phenotype. After 1 h of incubation with either *L. reuteri* LMG P-27481, *L. reuteri* DSM 17938 or *L. rhamnosus* ATCC 53103, the cells were washed thoroughly, the medium was changed to one containing antibiotic and 23 h later the cytokine released was quantified by ELISA. The three lactobacilli tested induced IL-10 and IL-12p70 release, although with a different profile (Figures 4A,B). Both *L. reuteri* strains were more effective than *L. rhamnosus* ATCC 53103 in stimulating cytokine secretion. Moreover, *L. reuteri* LMG P-27481 appeared more efficient than *L. reuteri* DSM 17938 in simulating IL-10 secretion. The anti-inflammatory index, calculated as the IL-10/IL-12p70 ratio, revealed that viable *L. reuteri* LMG P-27481 was the most effective strain in inducing an anti-inflammatory response in the DCs (Figure 4C). The same experiments were also conducted after the DCs were challenged with *Salmonella*. Overall, all the probiotic strains were significantly less efficient than *Salmonella* in inducing cytokine release (data not shown).

### *L. reuteri* LMG P-27481 Efficiently Metabolizes Lactose

The ability to counteract or at least mitigate the negative effects of various etiological agents of a chemical and/or microbial origin that are able to cause diarrhea is a highly desirable feature for a probiotic strain. Since lactose intolerance is one of the most common causes of diarrhea, probiotic strains were tested for their ability to hydrolyze lactose and thus, potentially, to limit the symptoms associated with lactose intolerance. Two well-known probiotic strains, *L. reuteri* DSM 17938 and *L. rhamnosus* ATCC 53103, were selected as our reference of comparison with the LMG P-27481 strain tested.

**FIGURE 5:**
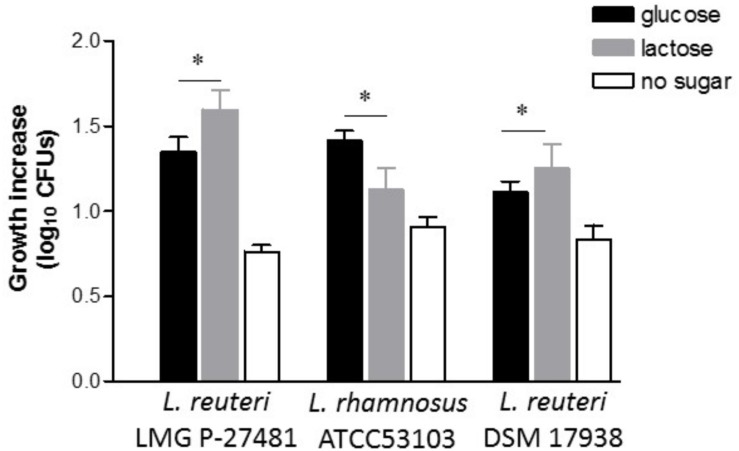
*Lactobacillus reuteri* LMG P-27481 preferentially utilizes lactose as a carbon source. The effect of the presence of only glucose (black bars), only lactose (gray bars) or no sugars (open bars) as carbon sources, on growth performance of *L. reuteri* LMG P-27481, *L. reuteri* DSM 17938 or *L. rhamnosus* ATCC 53103, was calculated as growth increase in log10 CFUs. Bacteria were incubated for 18 h with 1% of each type of sugar and growth was recorded by decimal counts, (*n* = 3 replicates for each condition). Data are presented as mean ± SD. * indicates *P* < 0.05 versus growth in MRS containing glucose.

Bacterial growth in the presence of glucose or lactose was compared. *L. reuteri* DSM 17938 seemed more active in the presence of lactose in the growth medium, indicating its ability to vigorously metabolize this carbon source. *L. rhamnosus* ATCC 53103 was instead clearly more active in presence of glucose with respect to lactose in the culture medium (Figure 5). The LMG P-27481 strain produced in the end the most significant growth in the presence of lactose with respect to the other strains (Figure 5). All strains tested showed a negligible growth in MRS broth without sugars (Figure 5).

### *L. reuteri* LMG P-27481 Antagonizes Pathogen Growth *in vitro*

**FIGURE 6:**
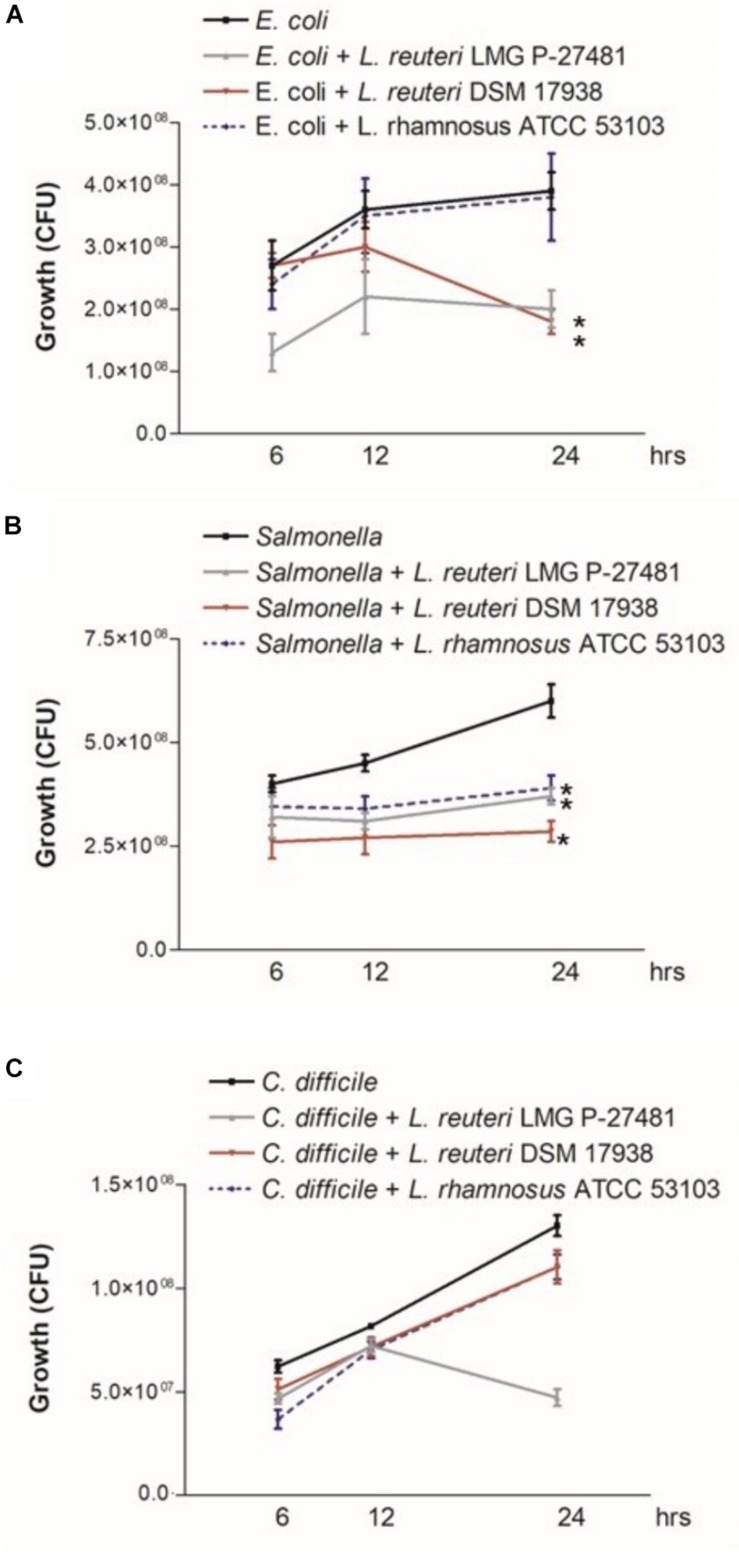
*Lactobacillus reuteri* LMG P-27481 inhibits pathogen growth *in vitro*. MRS broth was inoculated with 10^7^ CFU/ml of the strain and incubated in anerobiosis for 24 h. MRS conditioned broths were sterile filtered, pH adjusted to 7 and inoculated at 37°C with either *E. coli* (10^7^ CFU/ml) **(A)**, *Salmonella* (10^7^ CFU/ml) **(B)** or *C. difficile* (10^7^ CFU/ml) **(C)**. Aliquots of culture were collected after 6, 12 or 24 h and live bacteria quantified by seeding on proper Agar medium incubated at 37°C in aerobiosis for *E. coli, Salmonella* and anaerobiosis for *C. difficile*. Data are reported as mean ± SD on values obtained on three different determinations. *P < 0.05 versus not treated pathogen.

Intestinal pathogens, such as *E. coli*, *Salmonella* and *C. difficile*, were inoculated into supernatants of the probiotic strains tested and the inhibitory effects were verified. Figure 6 illustrates the growth patterns of the pathogens 6, 12, and 24 h following incubation in the probiotic supernatants. Although the probiotics tested were unable to significantly affect the growth of *Salmonella*, they all, however, slightly reduced pathogen growth as early as 6 h after incubation (0.2–0.3 log10 CFUs reduction compared to the control). *L. reuteri* DSM 17938 was the most effective in reducing *Salmonella* growth 24 h after incubation. Similarly, both *L. reuteri* strains hindered *E. coli* growth 6 h after incubation, and growth inhibition was maintained for the 24 h following co-incubation. *L. rhamnosus*, instead, did not modify the pathogen’s growth pattern. More interestingly, *L. reuteri* LMG P-27481 significantly inhibited *C. difficile* growth 24 h after incubation as the pathogen growth was reduced by more than 0.5 log10 CFUs. All the other strains produced a growth increase similar to that in the controls.

### *L. reuteri* LMG P-27481 Antagonizes Rotavirus in the *in vitro* Infection Model

The probiotics were then tested to verify their ability to protect human gut cells from Rotavirus infection. Two different experimental designs were used to mimic the probiotics’ potential protective action: in the first case by pre-treating HT-29 cells monolayers with probiotics before exposure to Rotavirus, and in the second by co-incubating probiotics and Rotavirus with human enterocytes. All the strains tested showed inhibitory effects against Rotavirus infection *in vitro*. The *L. rhamnosus* ATCC 53103 showed the highest inhibitory activity in the pre-treatment and competition protocols (86 and 91%, respectively) The *L. reuteri* DSM 17938 (50 and 89%, respectively) and *L. reuteri* LMG P-27481 (66 and 79%, respectively) also significantly reduced the copies of Rotavirus produced following the infection of the human intestinal cells (Table 5).

**TABLE 5:** The average number of copies of viral genome in the presence of the probiotics tested during the study (raw data expressed as DNA copies/5 × 10^5^ cells ± SEM) in the two experimental designs.

Probiotic	N. copies viral genome	
	Pre-treatment	Competition
Rotavirus alone	4371 ± 660 × 10^3^	4630 ± 720 × 10^3^
*L. reuteri* DSM 17938	2198 ± 450 × 10^3^*	531 ± 85 × 10^3^*
*L. reuteri* LMG P-27481	1528 ± 390 × 10^3^*	1012 ± 410 × 10^3^*
*L. rhamnosus* ATCC 53103	627 ± 90 × 10^3^*	421 ± 83 × 10^3^*

*p < 0.05 versus Rotavirus alone.

### *L. reuteri* LMG P-27481 Protects From *C. difficile* Infection *in vivo*

**FIGURE 7:**
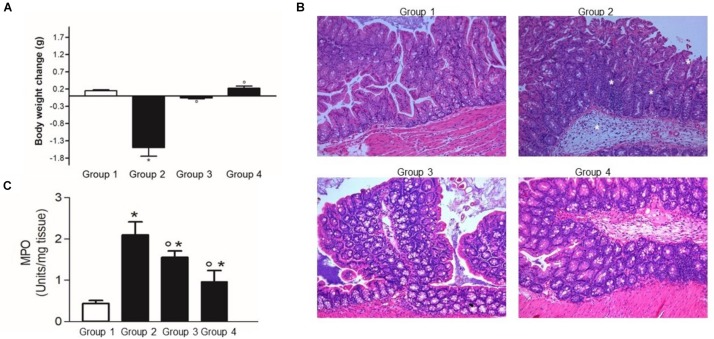
*Lactobacillus reuteri* LMG P-27481 reduces the severity of *C. difficile* colitis in a murine model. The mice were randomly divided in four experimental groups (Figure 1). Five days after intragastric (IG) challenge with *C. difficile* (10^5^ CFU) the animals were sacrificed, and the colon content collected. **(A)** Body weight was measured at baseline (IG challenge) and every day for the following 5 days. Weight change was calculated as the variation in weight at sacrifice compared with that at the baseline. **(B)** Full thickness colonic specimens were fixed in 4% formalin, paraffin embedded and 8 μm thick sections stained with H&E. Tissue edema, inflammatory infiltrate and mucosal ulcers were evaluated and indicated with * in the Group 2 image. **(C)** Thickness colonic specimens were homogenized and used to quantify MPO activity as a marker of neutrophils infiltration. Data are reported as mean ± SD on values obtained on six animals for each experimental group. *P < 0.01 versus group 1 and °*P* < 0.05 versus group 2.

Following the challenge with *C. difficile*, mice with no probiotic supplementation (group 2) showed a significant body weight loss over the next 5 days (Figure 7A). As expected, at the histological examination the mice challenged with *C. difficile* showed a significant inflammatory infiltrate with massive edema and loss of epithelial cells in the colonic mucosa (Figure 7B). Indeed, myeloperoxidase (MPO) activity in colonic mucosa, a marker of neutrophils infiltration, was significantly higher in Experimental group 2 with respect to the control mice (Figure 7C).

**FIGURE 8:**
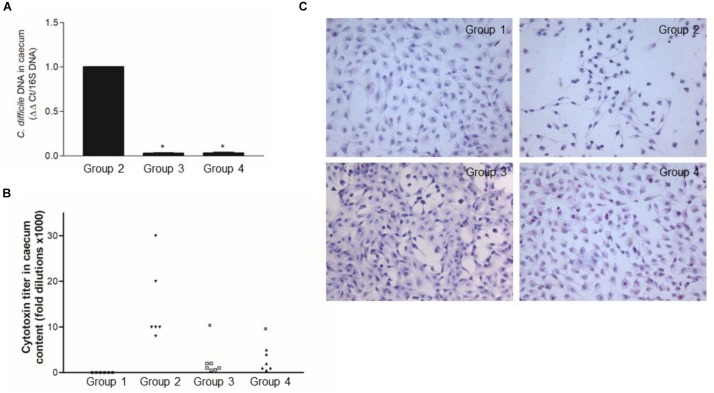
*Lactobacillus reuteri* LMG P-27481 reduces colonization in an antibiotic-induced *C. difficile* murine model. The mice were randomly divided into four experimental groups (Figure 1). Five days after intragastric (IG) challenge with *C. difficile* (10^5^ CFU) the animals were sacrificed, and the caecum content collected. **(A)** Total DNA was extracted, and *C. difficile* specific DNA was quantified by qPCR. The data were quantified by the ΔΔCt method using 16S rDNA as the reference gene. **(B)** The caecal content was centrifuged, the clear supernatant sterile filtered and added to Vero cells monolayers to determine cytotoxicity. **(C)** Serial 1:2 dilutions of caecal content were added to Vero cells monolayers; the titer of *C. difficile* toxins was defined as the highest dilution causing >50% rounding 24 h after incubation. Data are reported as mean ± SD on values obtained on six animals for each experimental group. *P < 0.05 versus group 2.

By seeding the caecal content on selective media, *C. difficile* was isolated from the caecal content of the mice challenged with the pathogen, but not from the control group (data not shown). qRT-PCR uncovered a low ΔΔCt value only in the mice challenged with the pathogen confirming that *C. difficile* was present in those mice (Figure 8A). Finally, there was a high cytotoxin titer (>50% Vero cells rounding at 10^–4^ dilution) in the caecal content of the mice challenged with *C. difficile* (Figure 8B).

The *L. reuteri* LMG P-27481 supplementation effectively reduced *C. difficile* colonization and colitis (Figures 7, 8), and it significantly diminished *C. difficile* induced body weight loss following pathogen challenge (Figure 7A). Moreover, histologic analysis and quantification of MPO activity demonstrated that it significantly reduced the inflammatory damage of the colonic mucosa (Figures 7B,C). Likewise, the amount of *C. difficile* DNA as well as toxins in the caecal content was significantly diminished in Experimental Groups 3 and 4 with respect to Group 2 (Figures 8A–C).

## Discussion

Lactobacilli are important members of the gut microbiota community that are able to influence host health through a variety of mechanisms (Oh et al., 2010). *L. reuteri*, a separate species within the *Lactobacillus* species, was chosen for isolation because it has been shown to exhibit probiotic efficacy and to confer broad-spectrum protection against disease in humans (Casas and Dobrogosz, 2000). Beyond its ability to survive the hostile conditions of the gastrointestinal environment including the high pH level of gastric juices and to tolerate bile salts, it shows resistance to antibiotics. It is, in addition, able to metabolize lactose, meaning that it can potentially alleviate the symptoms of intolerant subjects. Like some control strains (*L. reuteri* DSM 17938 and *L. rhamnosus* ATCC 53103), *L. reuteri* LMG P-27481 has been shown to produce a marked inhibitory effect on several pathogens (*E. coli*, *Salmonella* and Rotavirus) in different *in vitro* models. But as opposed to the control strains, *L. reuteri* LMG P-27481 has also been shown to diminish *C. difficile* growth *in vitro* and to significantly reduce *C. difficile* colonization and inflammatory colitis in mice.

Its safety, effectiveness, and genetic stability were investigated by means of genome sequencing, which uncovered the presence of two amino acid sequences similar to the mucus-binding MUB domains as well as a sequence coding for the typical N-terminal signal peptide (YSIRK motif) targeting the protein for secretion, and a C-terminal sortase recognition site (LPxTG), targeting the protein for covalent attachment to the peptidoglycan layer on the outside of the bacterial cell (Sengupta et al., 2013). As described by Etzold et al. (2014) and Bath et al. (2005), our experiments showed that the strain carried five sequences with a significant homology to mucus-binding proteins, cell wall anchor domains, and signal peptides characterizing the surface-exposed adhesins from *L. reuteri*. Moreover, the presence of putative EPS-coding sequences, which are known to interfere with the adhesion of enteropathogenic *E. coli* (EPEC) to pig erythrocytes, was also confirmed in the LMG P-27481 genome (Chen et al., 2014). *L. reuteri* is, in fact, one of the lactobacilli species known to produce EPS, whose chemical features seem to depend on the type of sugar substrate metabolized by the strain. Wang et al. (2010) demonstrated that EPS from *L. reuteri* was able to interfere with the *in vitro* adhesion ability of EPEC to pig erythrocytes (hemagglutination). Peptidoglycan hydrolases and EPS production could enhance the strain’s potential to exert antimicrobial activities against gut pathogens. Our experiments confirmed the safety of the LMG P-27481 strain because they demonstrated that sequences of well-known virulence genes were absent. The unforeseen finding regarding the anti-inflammatory potential of the LMG P-27481 strain that emerged here was the higher stimulation of IL-10 cytokine with respect to IL-12p70, as demonstrated by the resulting anti–inflammatory index (IL-10/IL-12 ratio). The differences in the expression of key markers of DC activation and polarization for the *L. reuteri* DSM 17938 and LMG P-27481 and *L. rhamnosus* ATCC 53103 strains may reflect putative synergy between strains possessing complementary beneficial features (Mileti et al., 2009). That synergy, or putative antagonism, should be carefully tested using a scientific approach, for example, by comparing the efficacy of single pure strains versus blended ones in treating diarrhea caused by different physio-pathological mechanisms.

*Lactobacillus reuteri* LMG P-27481 significantly reduced *C. difficile* growth *in vitro*, and, following oral challenge in mice, it reduced colonization of pathogen and colitis *in vivo*. In the authors’ opinion, the probiotic’s beneficial effects in the *C. difficile* colitis setting depended on the strain’s anti-microbial as well as anti-inflammatory action. It is well known that all stress or inflammatory mechanisms have a detrimental effect on gut barrier integrity, and other exacerbations induced by cyclic mechanisms disrupt the gut barrier function (Marjoram et al., 2015; Gil-Cardoso et al., 2016; Cui et al., 2017). The probiotic’s ability to induce anti-inflammatory effects in dendritic cells may have contributed to reducing inflammatory mucosal damage given the active role of these cells during *C. difficile* infection (Ausiello et al., 2006; Lee et al., 2009).

The inhibitory effects noted during our *in vitro* assays demonstrated that LMG P-27481 supplementation was able to significantly reduce *in vivo C. difficile* intestinal colonization. The reduced cytotoxic load in the colon lumen observed in the LMG P-27481-supplemented mice could have been the direct result of the probiotic’s inhibitory action against the pathogen. Since *C. difficile* toxins mediate colonic inflammatory damage (Abt et al., 2016), the reduced toxic load could have ameliorated the clinical outcome.

The mechanisms underlying the *in vivo* efficacy of *L. reuteri* LMG P-27481 observed require further investigation, but some observations can be made with regard to our genome analysis. The study, in fact, uncovered some distinctive genetic features associated to the presence of a unique pool of traits with respect to other *L. reuteri* strains whose genomes are publicly available. *L. reuteri* LMG P-27481 can adhere to and interact with gut epithelial cells and host cells and enhance epithelial barrier function; it also has putative but reliable beneficial effects on impaired gut mucosal functions. Moreover, peptidoglycan hydrolases and EPS production could further support the strain’s antimicrobial activities against gut pathogens.

## Conclusion

Taken together, the results of our work characterizing a new probiotic lactobacillus lead to the conclusion that *L. reuteri* LMG P-27481 represents a promising strain by virtue of its ability to adhere to human enterocytes and to stimulate anti-inflammatory cytokine secretion. After an initial assessment of the probiotic’s unique properties carried out following conventional protocols and international guidelines, the strain was subjected to a battery of assays leading to target-selection and characterization. The probiotic not only showed conventional probiotic features, which are essential for its successful activity, but also the ability to counteract the growth and colonization *in vitro* and *in vivo* of *C. difficile*. Further studies are warranted given the strain’s efficacy even in those cases in which it is administrated in association with antibiotic therapy. Its clinical application to treat *C. difficile* infection and forms of diarrhea caused by other conditions (i.e. lactose intolerance) also warrant further investigation.

## Data Availability Statement

The datasets generated for this study can be found in the WHOJ00000000.

## Ethics Statement

The strain involving human subjects was isolated following the written informed consent of the parents. Approval by an Ethical Committee was not required by local legislation at the time the strain was isolated. The animal study was carried out in accordance with the principles of the Basel Declaration and the recommendations of the Animal Care and Use Ethics Committee of the University of Padua under license from the Italian Ministry of Health; they were in compliance with national and European guidelines for handling and use of experimental animals. Experimental protocols were approved by the Animal Care and Use Ethics Committee of the University of Padua under license from the Italian Ministry of Health.

## Author Contributions

VS, FU, GB, AP, PB, IC, and ME performed the experiments. LB, LR, GM, PB, IC, and ME designed and interpreted the experiments. VS, FU, IC, and ME wrote the manuscript. LM critically reviewed the manuscript.

## Conflict of Interest

LB was employed by the Moviscom S.r.l. company that funded the study and owns the strain. GM and LR are employed by the Nòos S.r.l., the company that hold the license for the strain’s commercialization. ME, FU, and VS are employed by the AAT S.r.l. company, the contract laboratory that performed the assays. The remaining authors declare that the research was conducted in the absence of any commercial or financial relationships that could be construed as a potential conflict of interest.
